# MitoSegNet: Easy-to-use Deep Learning Segmentation for Analyzing Mitochondrial Morphology

**DOI:** 10.1016/j.isci.2020.101601

**Published:** 2020-09-29

**Authors:** Christian A. Fischer, Laura Besora-Casals, Stéphane G. Rolland, Simon Haeussler, Kritarth Singh, Michael Duchen, Barbara Conradt, Carsten Marr

**Affiliations:** 1 Fakultät für Biologie, Ludwig-Maximilians-Universität Munich, Planegg-Martinsried, Munich, 82152 Bavaria, Germany; 2 Centre for Integrated Protein Science, Ludwig-Maximilians-University, Planegg-Martinsried, Munich, 82152 Bavaria, Germany; 3 Institute of Computational Biology, Helmholtz Zentrum München – German Research Center for Environmental Health, Ingolstädter Landstr. 1, 85764 Neuherberg, Germany; 4 Department of Cell and Developmental Biology, Division of Biosciences, University College London, London WC1E 6AP, UK

**Keywords:** Cell Biology, Bioinformatics, Automation in Bioinformatics, Artificial Intelligence

## Abstract

While the analysis of mitochondrial morphology has emerged as a key tool in the study of mitochondrial function, efficient quantification of mitochondrial microscopy images presents a challenging task and bottleneck for statistically robust conclusions. Here, we present Mitochondrial Segmentation Network (MitoSegNet), a pretrained deep learning segmentation model that enables researchers to easily exploit the power of deep learning for the quantification of mitochondrial morphology. We tested the performance of MitoSegNet against three feature-based segmentation algorithms and the machine-learning segmentation tool Ilastik. MitoSegNet outperformed all other methods in both pixelwise and morphological segmentation accuracy. We successfully applied MitoSegNet to unseen fluorescence microscopy images of mitoGFP expressing mitochondria in wild-type and *catp-6*^ATP13A2^ mutant *C. elegans* adults. Additionally, MitoSegNet was capable of accurately segmenting mitochondria in HeLa cells treated with fragmentation inducing reagents. We provide MitoSegNet in a toolbox for Windows and Linux operating systems that combines segmentation with morphological analysis.

## Highlights

• MitoSegNet is a deep learning model for quantifying mitochondrial morphology
• MitoSegNet outperforms other mitochondria segmentation methods
• MitoSegNet was used to identify previously unreported morphological differences
• MitoSegNet successfully segments mitochondria in HeLa cells

## Graphical Abstract

**Figure:**
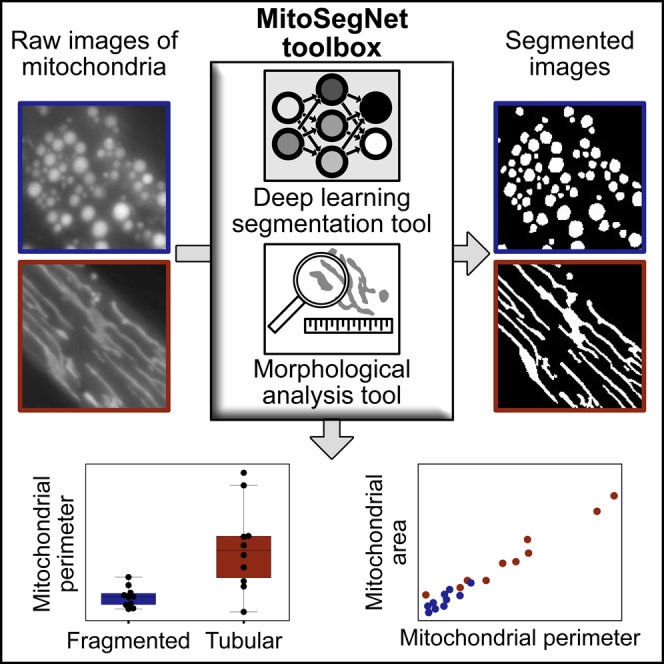

## Introduction

Cellular organelles are integral to eukaryotic cells and their functions. One organelle that has always been of particular interest is the mitochondrion, which plays an essential role in several metabolic pathways including that of Adenosine triphosphate (ATP). Mitochondria are often represented as static, bean-shaped organelles but actually form highly dynamic ‘tubular’ networks that often undergo changes in distribution and morphology (Tilokani et al., 2018; Chan, 2020). The steady-state morphology of mitochondria in a cell is a result of a balance between two opposing processes, mitochondrial fusion and fission. Changes in this balance result in changes in mitochondrial morphology. It has been shown that changes in morphology allow mitochondria to respond to metabolic or environmental stresses, while maintaining homeostasis (Tondera et al., 2009; Rolland et al., 2013; Wai and Langer, 2016). While partially damaged mitochondria can be rescued by exchanging their contents with functional mitochondria through mitochondrial fusion, mitochondrial fission enables the removal of damaged mitochondria and can also facilitate apoptosis during increased levels of cellular stress (Pernas and Scorrano, 2016). Mitochondrial fusion and fission are regulated by a conserved family of dynamin-related GTPases and have been well studied in *Caenorhabditis* elegans (van der Bliek et al., 2017). In *C. elegans*, the membrane anchored dynamin-related GTPases FZO-1^MFN^ and EAT-3^OPA1^ are required for the fusion of the outer- and inner mitochondrial membranes, respectively. The loss of function of either of these two proteins results in mitochondria with a ‘fragmented’ morphology (Breckenridge et al., 2008; Ichishita et al., 2008; Kanazawa et al., 2008; Tan et al., 2008; Rolland et al., 2009).

Mitochondrial fission in *C. elegans* is mediated by DRP-1^DRP−1^, a cytosolic dynamin-related GTPase. Depletion of DRP-1 has been shown to result in mitochondria with an ‘elongated’ morphology (Labrousse et al., 1999). Mutations in the human orthologs of the genes encoding these proteins have been associated with several diseases, including neurodegenerative diseases (Chan, 2020). For this reason, understanding mitochondrial fusion and fission is not only an important basic biological question but is critical for our ability to understand the pathology of these diseases and to develop novel therapeutics to treat them. However, such studies have been hindered by the fact that is difficult to assess mitochondrial morphology in different genetic backgrounds or physiological conditions in an unbiased and quantitative manner. Specifically, the diversity of shapes among mitochondria (elongated, fragmented, tubular, as well as ‘mixed’ morphologies) poses a challenge to the automated quantification of mitochondrial morphology. For this reason, researchers often resorted to the use of a simple qualitative assessment of mitochondrial morphology. As a result, subtle differences in morphology and, hence, phenotypes are often not detected. To analyze mitochondrial morphology, for example, in *C. elegans*, mitochondria are labeled using either a mitochondria-specific fluorescent dye (such as TMRE) or a transgene expressing a mitochondrial-targeted GFP (mitoGFP) (Regmi and Rolland, 2017) and a fluorescent microscopy image is acquired. In order to quantify the mitochondrial morphology in an automated and unbiased manner, the next critical step is to segment the objects in the fluorescent image. The simplest form of image segmentation is thresholding, which is only successful if features are well separated and their intensities vary considerably from the background (Torborg and Feller, 2004). These requirements are seldom met in live cell imaging due to autofluorescence, noise or fluctuating intensities. Thresholding segmentation can be improved through the prior application of feature enhancement algorithms based on intensity derived features, such as the Difference-of-Gaussians (DoG), Determinant of Hessian (Sato et al., 1998) or Laplacian-of-Gaussian (LoG), which are also known as blob detectors. DoG is used to enhance the visibility of edges by removing high frequency information but at the cost of reducing the overall image contrast, while LoG is useful for detecting edges that appear at different image scales or degrees of focus (Marr and Hildreth, 1980; Lindeberg, 1994). Curvilinear structures (such as nerve fibers or blood vessels) can be segmented using the eigenvalues of a Hessian matrix, with which one can calculate the object curvature. There are a many other methods available used in segmentation workflows, such as morphological filtering (dilation, erosion, etc.), region accumulation (watershed transform), deformable model fitting (active contour model) and machine learning (k-means clustering, random forest, etc.) (Meijering, 2012). Most of these methods can now be implemented by biologists through free and opensource tools such as Fiji (Schindelin et al., 2012), CellProfiler (McQuin et al., 2018) or Ilastik (Berg et al., 2019).

All of these segmentation methods have shown varying degrees of success depending on the images they were supposed to segment (de Boer et al., 2015; Li et al., 2015; Akram et al., 2017; Berg et al., 2019). However, with rising image complexity as well as a decreased signal-to-noise ratio, most of the methods perform poorly. For such cases, the only option in the past was manual segmentation, which is highly laborious and introduces a varying degree of bias on each labeled image. With the recent emergence of deep learning and in particular the development of convolutional neural networks (CNNs) (LeCun et al., 1989; Krizhevsky et al., 2012) automated approaches that perform these tasks with human accuracy have become available. CNNs were inspired by the research of Hubel and Wiesel on the primary visual cortex of cats (Wiesel and Hubel, 1963). CNN's can classify data based on convolution and pooling operations. Convolution describes the extraction of features from an image by sliding filters across the image and generating feature maps. Pooling reduces the dimensionality of each feature map, while retaining the most important information. It also reduces the number of network parameters, prevents overfitting, and makes the network invariant to small distortions in the input image (Scherer et al., 2010). Through the successive and repetitive application of convolution and pooling, CNNs are capable of classifying highly complex images with great accuracy (Szegedy et al., 2014). To perform semantic segmentation, which is the assignment of a class label to each pixel, one must use a fully convolutional neural network (FCNN) (Long et al., 2014). A popular FCNN in the biological community is the U-Net that was specifically developed for biomedical image segmentation (Ronneberger et al., 2015). It has been successfully applied to many different biomedical image segmentation tasks and yields good results with only a few hand-segmented images (Chlebus et al., 2018; De Fauw et al., 2018; Stember et al., 2018).

In this study, we trained a U-Net, which we named Mitochondrial Segmentation Network (MitoSegNet), to learn how to segment mitochondria in adult *C. elegans* body wall muscle cells, compared its performance and tested its generalizability in biologically relevant applications that demonstrate *C. elegans* animals carrying a loss-of-function mutation in the gene *catp-6*^*ATP13A2*^ exhibit a previously unreported mitochondrial morphology phenotype. We also show that MitoSegNet can be successfully used to analyze mitochondrial morphology in HeLa cells.

## Results

### The MitoSegNet Model

**Figure 1:**
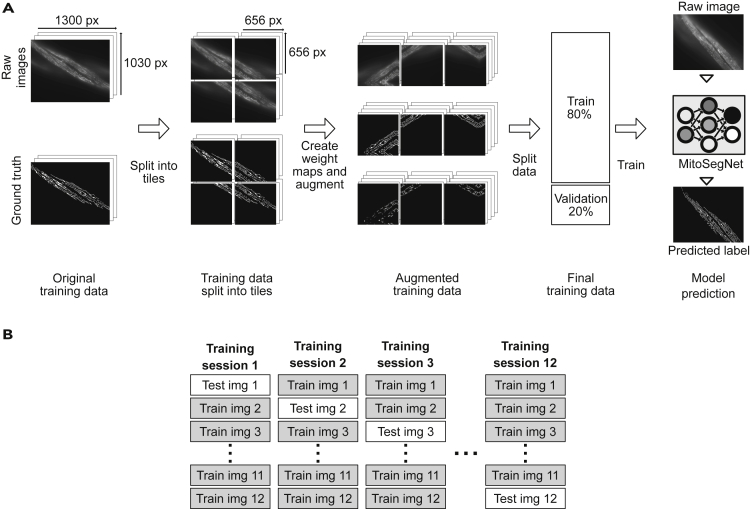
Training the MitoSegNet Model and Using It with the MitoS Tool (A) The original training data is comprised 12 raw images and the appendant hand-generated ground truth images. Each image is split into 4 overlapping tiles of equal length. For each tile, a weight map is generated and subsequently all three set of tiles (raw images, ground truth, and weight maps) are augmented 80 times, increasing the size of the training data to 3,840 image tiles. Prior to training, the augmented training data is split into training (80%) and validation data (20%). The pretrained MitoSegNet model can now be used to segment new images of mitochondria. (B) We performed a cross validation for which 12 separate MitoSegNet models were trained each with 11 images, excluding one image that was later used to test the prediction accuracy against other segmentation methods.

The MitoSegNet model was generated by training a modified U-Net with a training set of 12 1300 × 1030 pixel fluorescent microscopy, maximum-intensity projection images, depicting mitochondria in body wall muscle cells of adult *C. elegans* worms (mitochondria were visualized using a transgene expressing mitochondrial matrix-targeted GFP under the control of a body wall muscle-specific promoter (*P*_*myo3*_*::mitoGFP*)) (Figure 1A and Methods). Our U-Net modification entails the removal of dropout layers at the end of the contracting pathway and instead placing batch normalization layers after every convolutional layer prior to ReLU activation in the contracting pathway. This modification decreased the amount of necessary training time. Each image was split into 4 overlapping tiles. For each tile, 80 augmented copies were generated for training the model. A cross validation was performed to estimate the performance of the MitoSegNet on an unseen test set and to compare it against other segmentation methods (Figure 1B).

### Visual Comparison of Segmentation Performance

**Figure 2:**
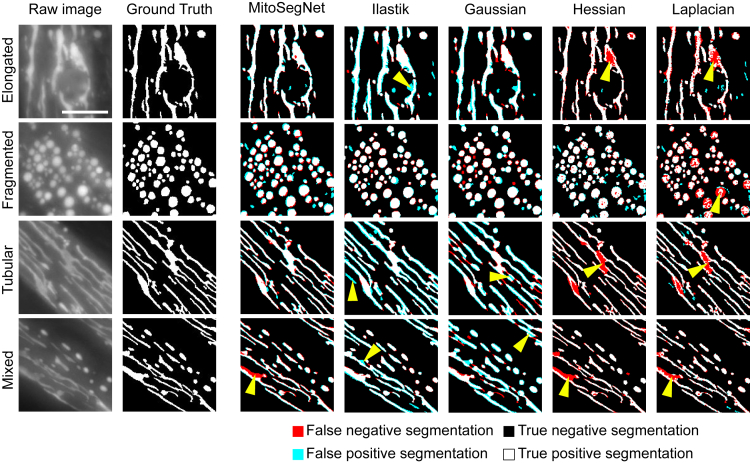
MitoSegNet Visually Outperforms Feature-Based Segmentation Approaches On the left side, four images of elongated, fragmented, tubular, and mixed *C. elegans* mitochondria and their respective ground truth are shown. The masks on the right show the results of MitoSegNet and the four segmentation methods applied to each image, displaying the false negative segmentation in red, the false positive segmentation in turquoise, the true negative segmentation as black, and the true positive segmentation as white. The yellow arrows indicate areas in which false segmentation occured. The scale bar is 5 μm.

To qualitatively evaluate the performance of the MitoSegNet, we compared the predicted segmentations against manually segmented ground truth in an unseen test set. The same procedure was repeated for four other segmentation methods. We considered three classical feature enhancement methods (Gaussian, Hessian, and Laplacian) followed by different thresholding algorithms, all implemented in ImageJ/Fiji. The fourth method is the machine-learning segmentation tool Ilastik (Kreshuk and Zhang, 2019). The Gaussian, Hessian, Laplacian, and Ilastik methods failed to consistently prevent false positive and/or false negative segmentation on all phenotypes (Figure 2). The Gaussian segmentation produced large sections of false positive predictions in the mixed and tubular phenotype (indicated by yellow arrows). The Hessian and Laplacian segmentation largely avoided false positive predictions but instead often failed to recognize mitochondria, resulting in false negative segmentations in the elongated, mixed, and tubular phenotype (and fragmented for the Laplacian segmentation) (Figure 2). The Ilastik-based segmentation produced only very little false negative predictions but like the Gaussian segmentation, predicted large amounts of false positives in all but the fragmented phenotype. The MitoSegNet segmentation drastically reduces the amount of false negative or false positive segmentation when compared to the other methods and yielded consistent results across all different phenotypes (Figure 2).

### Quantitative Comparison of Segmentation Performance

**Figure 3:**
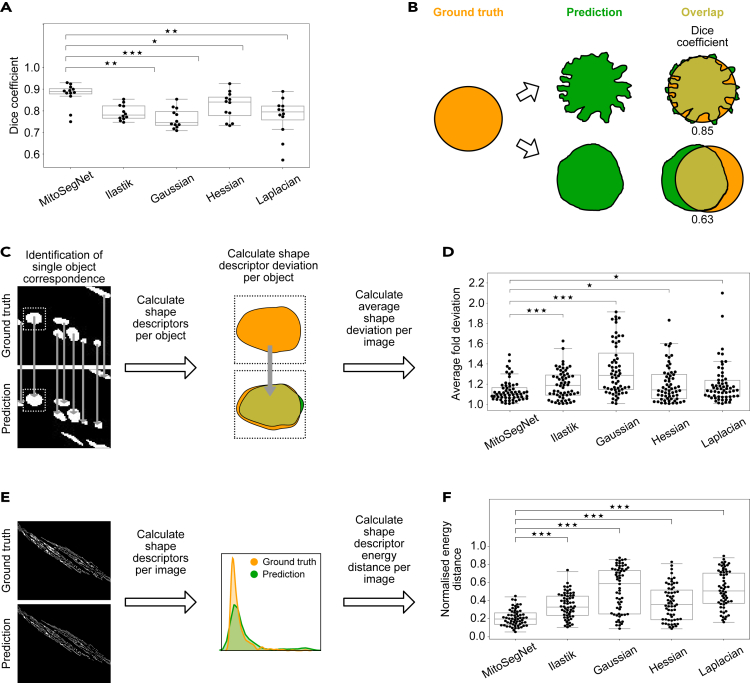
MitoSegNet's Pixelwise Accuracy Outperforms Non-Deep Learning Segmentation Methods (A) The average dice coefficient achieved with the MitoSegNet is significantly higher than with the four other segmentation approaches. The data was statistically evaluated by using the Kruskal-Wallis test followed by a Dunn's multiple comparisons test. (B) The dice coefficient is limited as a predictor of morphological segmentation performance: A dice coefficient close to 1.0 does not guarantee correct prediction of morphology. Contrary, a low dice coefficient does not rule out an accurate shape prediction. Ground truth segmentation is shown in orange, and the predicted segmentation in green. (C) To gain insight into how accurately the shape of ground truth objects is predicted, five object shape descriptors (area, eccentricity, aspect ratio, perimeter, and solidity) are compared by calculating the fold deviation. Predicted objects that correspond to more than one ground truth object (or vice versa) are excluded from this analysis. (D) The MitoSegNet shows the lowest average fold deviation between predicted and ground truth object shape descriptors. The data were statistically evaluated by first testing for normality using D'Agostino's K-squared test and then subsequently using the Kruskal-Wallis test followed by a Dunn's multiple comparisons test. N = 60. (E) To determine the total morphological prediction accuracy, the same five shape descriptors were measured for each image. The descriptor distributions in the ground truth and predicted images were statistically evaluated for differences by calculating the energy distances between predicted and ground truth distribution. The energy distances for each shape descriptor and image were normalized prior to statistical analysis. (F) The MitoSegNet shows the lowest normalized energy distance, statistically outperforming all other segmentation approaches. The data were first tested for normality using the D'Agostino's K-squared. After determining that all distributions were non-parametric, a Kruskal-Wallis test was used followed by a Dunn's multiple comparisons test. N = 60. ∗p < 0.05, ∗∗0.001 < p < 0.01, ∗∗∗p < 0.001 for (A), (D) and (F).

To compare the methods' performance more quantitatively, we evaluated the pixelwise segmentation accuracy using the dice coefficient (Taha and Hanbury, 2015). The MitoSegNet outperforms the feature-based and Ilastik-generated segmentations (Figure 3A) with a median dice coefficient of 0.89 and a lower and upper 95% confidence interval of 0.87 and 0.91 (N = 12) significantly (p = 5.11∗10^−5^, Kruskal-Wallis test). However, pixelwise accuracy as measured by the dice coefficient does not necessarily guarantee correct prediction of morphology (Figure 3B). Because segmented images in biology are often used for morphological quantification (de Boer et al., 2015; Abdolhoseini et al., 2019; Orozco-Fuentes et al., 2019), we assessed the morphological accuracy with two other approaches. The single object shape deviation per object was measured for five shape descriptors (area, eccentricity, aspect ratio, perimeter, and solidity) and averaged over 12 images (Figure 3C). The MitoSegNet with a median average fold deviation of 1.09 and a lower and upper 95% confidence interval of 1.07 and 1.12 (N = 60) outperforms all other methods in the accurate prediction of single object morphology (p = 7.4∗10^−10^, Kruskal-Wallis test) (Figure 3D). Because the single object shape deviation method does not consider false negative predictions, we also compared all segmented objects in ground truth and prediction. For each image and each of the five object descriptors, the energy distance between the ground truth and predicted distributions was calculated (Figure 3E). Due to the different value ranges among the descriptors the values were normalized prior statistical analysis. The MitoSegNet segmentation achieves a median normalized energy distance of 0.20 with a lower and upper 95% confidence interval of 0.16 and 0.23 (N = 60) and again statistically outperforms all other non-deep learning segmentation methods (p = 3.3∗10^−18^, Kruskal-Wallis test) (Figure 3F).

### Comparison of Mitochondrial Morphology between *Catp-6*^ATP13A2^ Mutant and Wild-type

**Figure 4:**
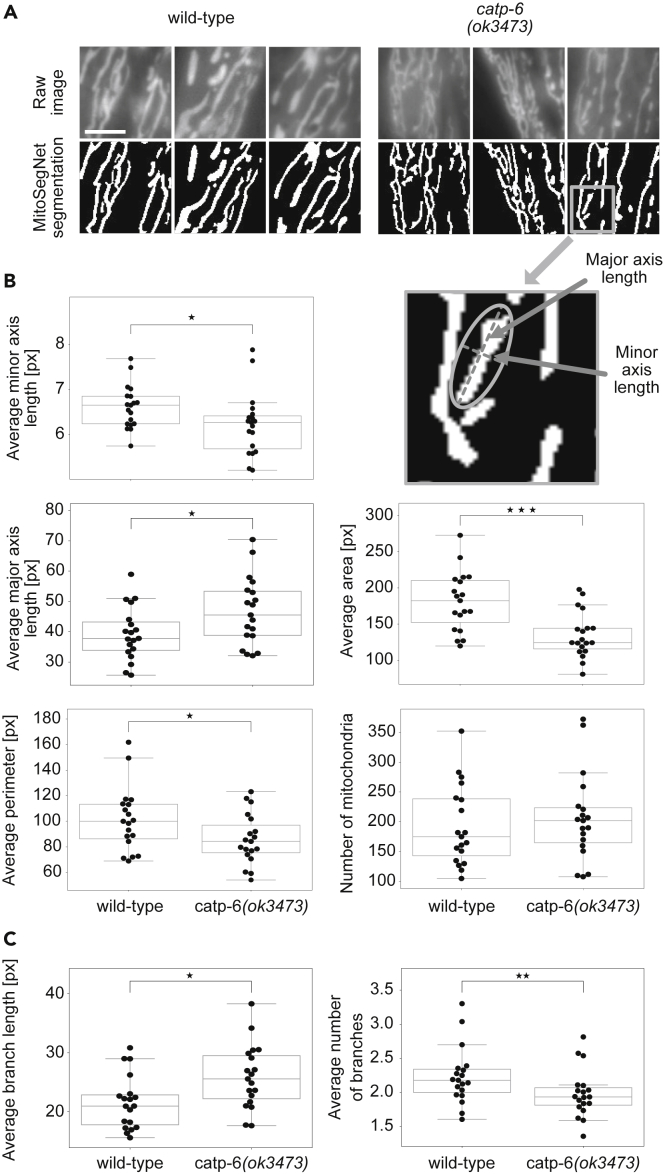
Wild-type and *Catp-6* Mutant Mitochondria Show Significant Morphological Differences (A) Visual comparison of *catp-6* mutant and wild-type mitochondrial morphology. Raw images are at the top and MitoSegNet model segmentations at the bottom. The scale bar is 3 μm. (B) Mitochondrial shape descriptor comparison. (C) Mitochondrial branch descriptor comparison. Average area, minor and major axis length (see scheme for explanation), perimeter, number of mitochondria, average branch length, and number of branches were measured in segmented images of wild-type and *catp-6* mutant mitochondria. ∗p < 0.05, ∗∗0.001 < p < 0.01, ∗∗∗p < 0.001 using the Mann-Whitney U test. N = 19.

**Figure 5:**
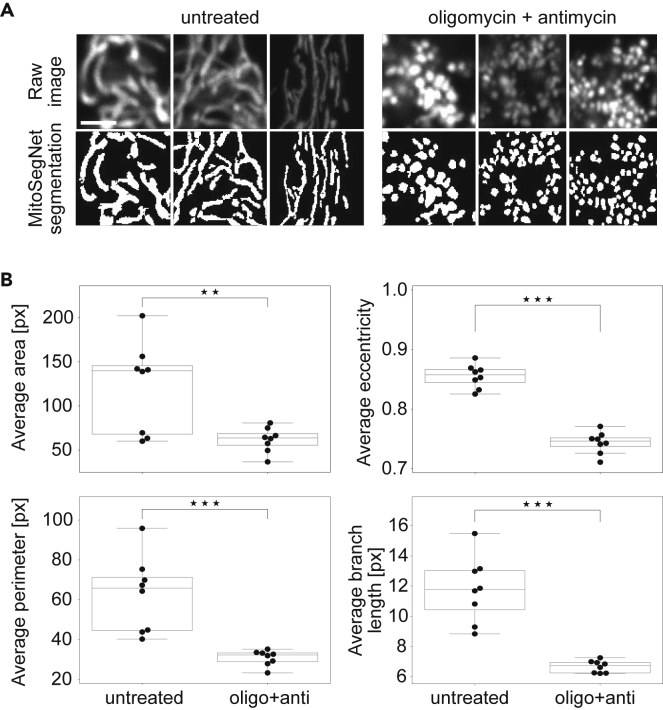
MitoSegNet Model Segmentation and Morphological Quantification Can Be Applied to Mitochondria of Mammalian Cells Comparing untreated HeLa cells and HeLa cells treated with oligomycin or antimycin for a duration of 3 h. (A) Visual comparison of untreated and treated mitochondrial morphology. Raw images are at the top and MitoSegNet model segmentations at the bottom. The scale bar is 2.5 μm. (B) Average area, eccentricity, perimeter, and branch length of mitochondria were measured in segmented images of treated and untreated mitochondria. ∗p < 0.05, ∗∗∗p < 0.001 using an independent two-sample t test. N = 8.

To evaluate the applicability of the MitoSegNet on a different, unseen set of images, we decided to use the MitoSegNet to determine whether a loss-of-function mutation of the gene *catp-6*^ATP13A2^, *ok3473* (hereafter referred to as *catp-6*(lf)), causes a mitochondrial morphology phenotype*. catp-6*^ATP13A2^ encodes a member of the family of P-type ATPases, which transport various compounds across membranes using ATP hydrolysis as energy source (Moller et al., 1996; Lambie et al., 2013; Anand et al., 2020). In addition, *catp-6*^ATP13A2^ is the human ortholog of ATP13A2, mutations of which leads to Kufor-Rakeb syndrome, a form of inherited juvenile-onset Parkinsons disease (Ramirez et al., 2006; Di Fonzo, Chien et al., 2007). No abnormal differences in mitochondrial morphology has so far been reported for the *catp-6*(lf). Consistent with this, upon brief visual inspection, no obvious differences in mitochondrial morphology are noticeable (Figure 4A). We applied the MitoSegNet (Figures 4A) to 19 fluorescence microscopy images of each genotype and subsequently analyzed the data with the MitoA tool (Figure 4B). Segmentation masks (Figure 4A) visually matched the raw images closely and subsequent quantification revealed a statistically significant morphological difference between mitochondria in wild-type and *catp-6*(lf) mutants. Compared to wild-type, mitochondria are significantly thinner and longer in *catp-6*(lf) mutants, as determined by the average minor (p = 0.047, independent two-sample t test) and major axis length (p = 0.029, independent two-sample t test) (Figure 4B). Furthermore, the average mitochondrial area is larger (p = 0.00039, independent two-sample t test) and the perimeter is longer (p = 0.043, independent two-sample t test) in wild-type compared to *catp-6*(lf) mutants (Figure 5B). Excessive mitochondrial fission (i.e. mitochondrial fragmentation) as a cause for these observations can be excluded since the numbers of mitochondria in wild-type and *catp-6*(lf) mutants are similar (p = 0.56, independent two-sample t test) (Figure 4B). Differences were also found in terms of mitochondrial branch morphology (Figure 4C). While the average mitochondrial branch length in *catp-6*(lf) mutants is larger than in wild-type (p = 0.01, independent two-sample t test), the average number of mitochondrial branches was found to be significantly smaller in *catp-6*(lf) mutants (p = 0.009, Mann-Whitney U test). Altogether these results indicate that *catp-6(ok3473)* causes a previously unreported mitochondrial morphology phenotype that cannot be detected by simple visual inspection.

### MitoSegNet Segmentation of Mitochondria in HeLa Cells

To test the generalizability of our model even further, we applied the MitoSegNet to segment 8 confocal microscopy images depicting mitochondria in HeLa cells (Figure 5A). The fragmentation of mitochondria in HeLa cells treated with oligomycin and antimycin for 3 hr was captured in the segmentation both visually and quantitatively. As expected, the average mitochondrial area is significantly larger in untreated cells compared to treated HeLa cells (p = 0.0068, independent two-sample t test) (Figure 5B). The average eccentricity is lower for the fragmented mitochondria compared to the untreated mitochondria, indicating a more circular shape (p = 1.32∗10^−8^, independent two-sample t test) (Figure 5B). The average perimeter distribution reflects a similar pattern as found for the area, showing the fragmented mitochondria to have a smaller perimeter on average (p = 0.00037, independent two-sample t test) (Figure 5B). The average branch length is also significantly smaller in the treated mitochondria compared to the untreated mitochondria (p = 1.30∗10^−5^, independent two-sample t test) (Figure 5B).

### The MitoS Segmentation and MitoA Analysis Tool

To enable non-experts, we implemented the MitoSegNet in an easy-to-use tool, the MitoS segmentation tool, a Python-based, standalone executable. MitoS can be executed in a basic mode, which utilizes the pretrained MitoSegNet for segmentation of mitochondria and allows us to easily apply the model without prior deep learning experience (Figure 1). We applied the MitoS image segmentation using the pretrained MitoSegNet on two different systems. For all cases 10 images of each 1300 × 1030 px size (8-bit) were segmented. The MitoS GPU segmentation was run using an NVIDIA GeForce GTX 960M and NVIDIA TITAN X and segmentation took 65 and 15 s, respectively. Segmentation using the MitoS CPU version was performed on an Intel(R) Core(TM) i7-6700HQ CPU and a system using four Intel(R) Xeon(R) CPU E5-2680 v4 processors and lasted 7.5 min and 65 s, respectively. The basic mode also includes a fine-tuning module, which allows us to optimize the pretrained on new images (Figure S1A). The MitoS advanced mode can be used if other structures besides mitochondria should be segmented or if the user wishes to build a self-configured deep learning segmentation model (Figure S1B). The MitoA analyzer is a separate Python-based, standalone executable tool that can be run after successful segmentation for quantification and visualization of potential morphological differences (Figure S3). It measures ten different morphological and three intensity-based features for each object and summary statistics for all object features per image are generated. The tables of two or multiple samples containing these summary statistics can then be subjected to hypothesis testing, visualization, and correlation analysis. The MitoS and MitoA tools require no installation and no prerequisite installations (such as frameworks), and they are available for both Windows and Linux.

## Discussion

We present MitoSegNet, a segmentation model that exploits the power of deep learning to address the challenging problem of accurate mitochondria segmentation. We show that the MitoSegNet outperforms feature-based, non-deep learning-based algorithms and that it is generalizable to unseen images from *C. elegans* and mammalian cells.

### MitoSegNet Model Segmentation Performance

While the superior visual and quantitative performance of the MitoSegNet model segmentation might not come as a surprise to researchers acquainted with the capabilities of deep learning-based segmentation, we believe these results to be interesting to researchers who commonly use feature-based segmentation methods. For accuracy evaluation, we did not rely only on pixel-based accuracy, as we found this to be an insufficient measure of morphological accuracy but extended our analysis. Our single object shape comparison as well as the calculation of energy distances for five different feature descriptors per image demonstrate that the MitoSegNet model segmentation yields the best morphological accuracy compared with commonly applied segmentation methods.

### Comparison of Mitochondrial Morphology between *Catp-6*^ATP13A2^ Mutants and Wild-type

The visual comparison of mitochondrial morphology in wild type and *catp-6*(lf) mutants did not reveal any obvious differences in morphology. In both cases, mitochondria appeared to be largely tubular. However, the quantitative analysis using the MitoSegNet revealed that average area, perimeter, and minor axis length of mitochondria in *catp-6*(lf) mutants are smaller than in wild-type. Since the average number of mitochondria per cell is the same as in wild-type, we concluded that *catp-6*(lf) causes a reduction in overall mitochondrial mass. This is consistent with observations of Anand et al., who found that oxygen consumption is lower in *C. elegans catp-6*(lf) mutants than in wild type (Anand et al., 2020). In mammalian cells, mutation of *ATP13A2* has been found to cause impairment of mitochondrial function and induce mitochondrial fragmentation (Gusdon et al., 2012; Ramonet et al., 2012). However, this is associated with increased mitochondrial mass due to inefficient autophagic clearance (Grünewald et al., 2012). The differences between the mutant phenotypes observed in *C. elegans* and mammalian cells is likely to reflect the multiple roles of ATP13A2 and CATP-6 transport substrates (polyamines) in maintaining mitochondrial function (van Veen et al., 2020). Further research on properties of *catp-6* deficient mitochondria, such as membrane potential or levels of reactive oxygen species, might uncover the cause for the observed differences in mitochondrial morphology.

### Application of the MitoSegNet to Mitochondria in HeLa Cells

Although the pretrained model was generated with standard fluorescence microscopy images, depicting mitoGFP-labeled mitochondria in *C. elegans*, the same pretrained model was able to generate visually accurate segmentations of mitoRFP labeled mitochondria in HeLa cells using a laser scanning confocal microscope. This demonstrates the high robustness and generalization capabilities of our pretrained MitoSegNet model and that it can be used for segmentation of mitochondria in organisms other than *C. elegans*. Furthermore, our MitoSegNet Analysis tool quantitatively confirmed the morphological differences of mitochondria between untreated HeLa cells and HeLa cells treated with oligomycin or antimycin.

### MitoSegNet Model Architecture

Although the MitoSegNet architecture is largely based on the U-Net, through testing various changes in the original architecture, we found that the validation dice coefficient as well as the validation loss improved upon removing the dropout layers and instead placing a batch normalization layer (Ioffe and Szegedy, 2015) after every convolution layer in the contracting pathway. Interestingly, a recent study found that the combined usage of batch normalization followed by dropout (forming an independent component layer) stabilized the training process, increased convergence speed, and improved the convergence limit (Chen et al., 2019). It would require further testing to find out if the usage of an independent component layer would improve the current MitoSegNet performance even further.

### MitoS and MitoA Tools

Most deep learning applications in the field of biological image segmentation were created for the purpose of 2D cell segmentation (Chen et al., 2017; Al-Kofahi et al., 2018; Falk et al., 2019; Kusumoto and Yuasa, 2019), while organelle-specific deep learning applications are scarce. Although most tools allow the user to retrain available 2D cell segmentation models to segment other biological structures of interest, this often requires computer science-related skills, such as familiarity with programming languages, shell interaction or knowledge on how to install various deep learning frameworks. One of the main motivations behind MitoSegNet and the MitoS and MitoA toolbox was to make deep learning segmentation accessible to researchers that do not have an extensive background in computer science or deep learning. The MitoS tool can be run without installation. The simple graphical user interface allows users to quickly navigate the MitoS and MitoA tools. The MitoS basic mode also comes with a fine-tuning module that allows researchers that would like to segment other organelles or images taken under different conditions than those used for training the MitoSegNet model. Since the subsequent step after segmentation is usually the analysis, we included the MitoA tool to save researchers the time to look up appropriate analysis tools and instead be able to quickly obtain potentially interesting insights.

### Conclusion

The MitoSegNet has been shown to outperform both conventional feature-based and machine-learning-based segmentation of mitochondria. The pretrained model can be easily applied to new 2D microscopy images of mitochondria through the usage of the MitoS tool, which is available for both standard and high-end Windows and Linux systems. Successfully segmented images of mitochondria can be subjected to quantification, statistical testing, and visualization with the MitoA tool.

### Limitations of the Study

The MitoSegNet model used for segmentation of images depicting fluorescently labeled mitochondria in *C. elegans* and HeLa cells was trained with 12 pairs of raw images and the appendant hand-labeled ground truth images. Although both the visual and quantitative segmentation accuracy was shown to be high in this study, there remains a bias which is based on the two annotators who generated 6 ground truth images each. To reduce the ground truth bias and increase the generalizability of the MitoSegNet model, more images labeled by different annotators can be added. Furthermore, image sections in which single mitochondria were not clearly distinguishable due to optical constraints of the microscope used to generate the image, made it difficult for the annotator to create labeled images that accurately represent the ground truth. This uncertainty introduced to the MitoSegNet model can be decreased by adding images recorded with higher resolution, thus reducing the sections in which such visually indistinguishable mitochondria exist.

### Resource Availability

#### Lead Contact

Further information and requests for resources should be directed to and will be fulfilled by the Lead Contact, Carsten Marr (carsten.marr@helmholtz-muenchen.de).

#### Materials Availability

Images used for training and testing the model are available upon request.

#### Data and Code Availability

The software documentation for the MitoS and MitoA tool can be found at https://github.com/mitosegnet. The MitoSegNet segmentation model, the MitoA analysis and MitoS segmentation tool (GPU/CPU) for Linux and Windows are available at https://zenodo.org/search?page=1&size=20&q=mitosegnet. The Python code used for generating the figures displayed in the manuscript is available at https://github.com/MitoSegNet/MitoSegNet_AccuracyTesting_Manuscript.

## Methods

All methods can be found in the accompanying Transparent Methods supplemental file.

## Supplemental Information

Document S1. Transparent Methods and Figures S1–S5
