# Supplementary material for: MitoSegNet: Easy-to-use Deep Learning Segmentation for Analyzing Mitochondrial Morphology
Source: iScience. 2020 Sep 29;23(10):101601. doi: 10.1016/j.isci.2020.101601 (PMC7554024; doi:10.1016/j.isci.2020.101601)
Supplement: Document S1. Transparent Methods and Figures S1–S5 [file mmc1.pdf]

iScience, Volume *23*

Supplemental Information

MitoSegNet: Easy-to-use Deep Learning Segmentation for Analyzing Mitochondrial Morphology

Christian A. Fischer, Laura Besora-Casals, Stéphane G. Rolland, Simon Haeussler, Kritarth Singh, Michael Duchen, Barbara Conradt, and Carsten Marr

Figure S1

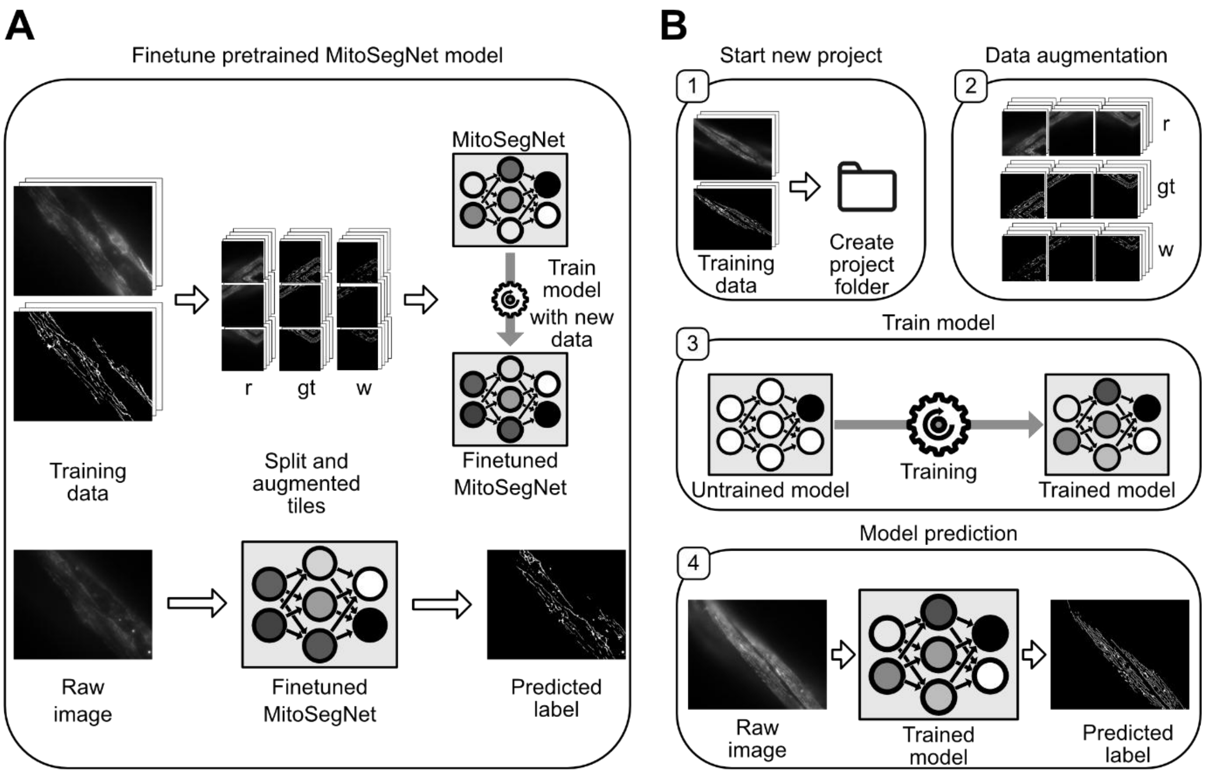

Figure S1: MitoS segmentation tool finetuning module and advanced mode workflow, Related to Figure 1 **(A)** The finetuning module enables the user to add new training data to finetune the existing MitoSegNet. This function automatically splits the new training data into tiles, performs augmentation (r: raw images, gt: ground truth images, w: weight map images) and the augmented data is then used to train the pretrained MitoSegNet. After completion of training, the finetuned MitoSegNet can be used for segmentation of new images using the prediction function. **(B)** The advanced mode allows the user to create a new deep learning segmentation model by adjusting network parameters. [1] Generation of a new project folder, in which all subsequent advanced functions will be carried out. [2] Data augmentation parameters can be specified and users can decide whether weight maps should be generated. [3] Model training allows the user to set the learning rate, batch size, class balance weight factor and if weight maps should be used. [4] Once the model has been trained the prediction function of the MitoS tool can be used to predict the segmentation on previously unseen images.

Figure S2

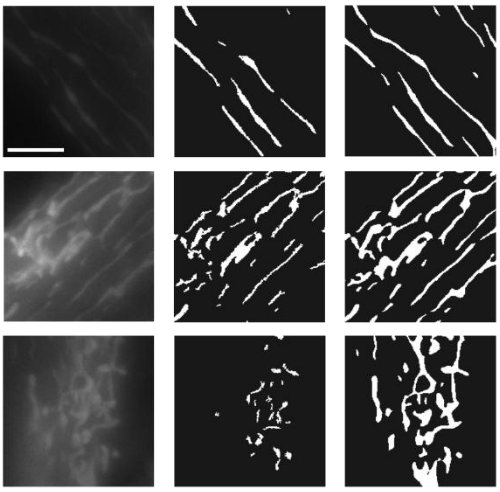

Figure S2: Finetuning MitoSegNet improves visual segmentation results, Related to Figure 2. Due to the usage of a non-integrated P_{myo-3}::mitoGFP reporter, the intensity of fluorescently labelled mitochondria appeared weaker and hence the MitoSegNet model failed to accurately segment these images. The input images are shown on the left, in the middle are the binary masks before finetuning and to the right the same masks after finetuning. By segmenting one image by hand and finetuning the pretrained segmentation model for 10 epochs, the visual segmentation results could be largely improved. The scale bar is 5µm.

Figure S3

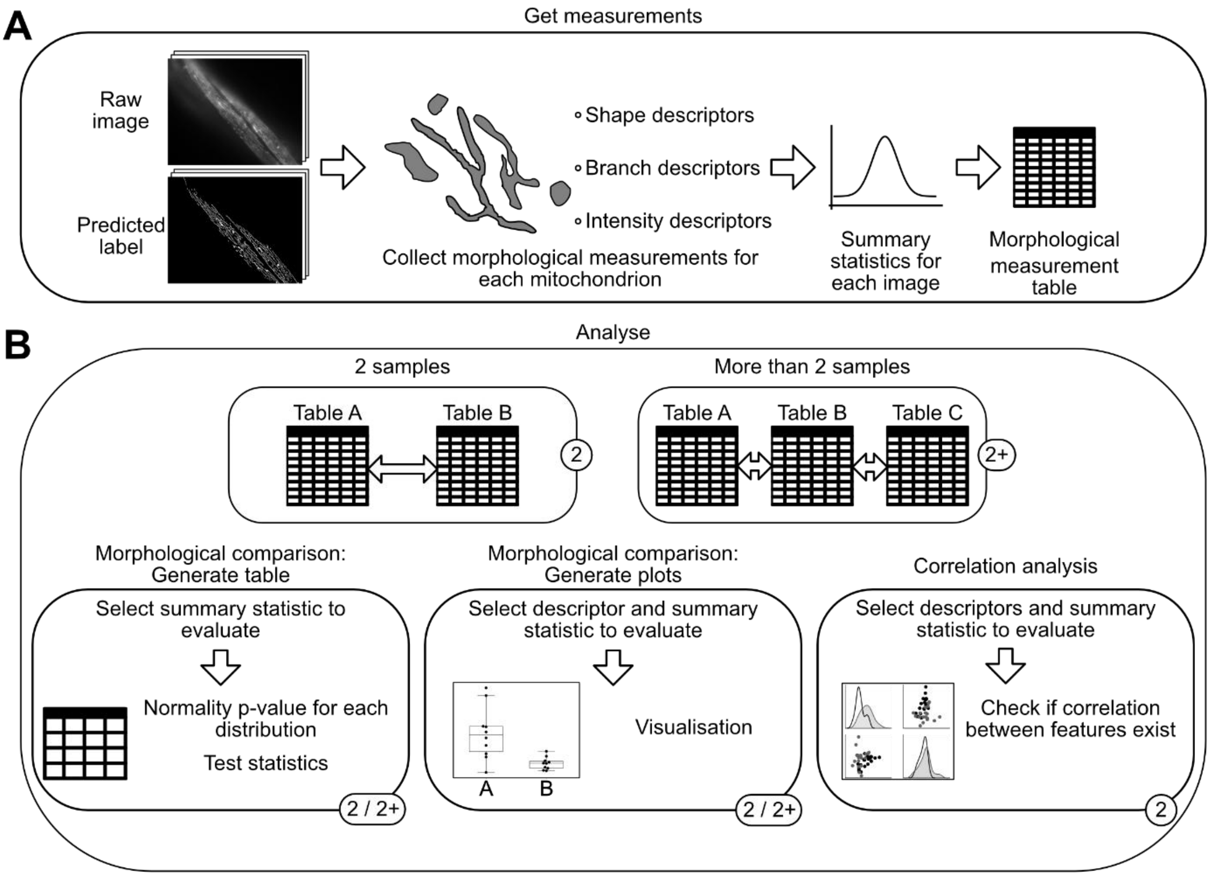

Figure S3: MitoA analyser tool workflow, Related to Figure 4 and 5. The two main functions of the MitoA tool are to get measurements or to analyse. To analyse images of interest, one has to first get the relevant measurements. **(A)** The "Get measurements" function uses images and their predicted labels to measure 6 different shape descriptors, 4 branch descriptors and the mean, maximum and minimum fluorescence intensity for each segmented object. The summary statistics are then saved to a measurements table. **(B)** Analysis can either be performed on only two samples (2) or more than two samples (2+). Subsequent statistical analysis and visualisation can be used in both cases but the correlation analysis is currently only implemented for the two sample comparison. The generate table function allows the user to select a summary statistic to evaluate (such as average, median or standard deviation) after which a table is generated in which sample distribution normality is tested and an appropriate statistical test is selected. The generate plots function lets the user select a feature descriptor and summary statistic to display as a boxplot. With the correlation analysis, up to 4 different feature descriptors from two samples can be visualised as scatterplots, including the display of the correlation coefficients.

Figure S4

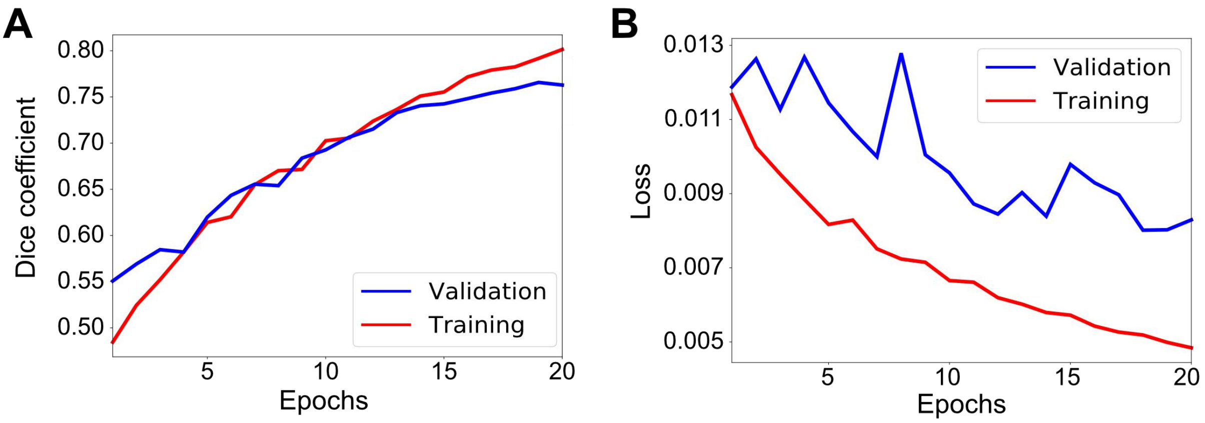

Figure S4: Training performance of final MitoSegNet trained on 12 images, related to Figure 1. (A) Training (red) and validation (blue) dice coefficient increase much like during cross validation and after epoch 15 discrepancy between training and validation dice coefficient begins to increase. **(B)** Training and validation loss decrease and reaches its minimum of 0.008 at 18 epochs.

Figure S5

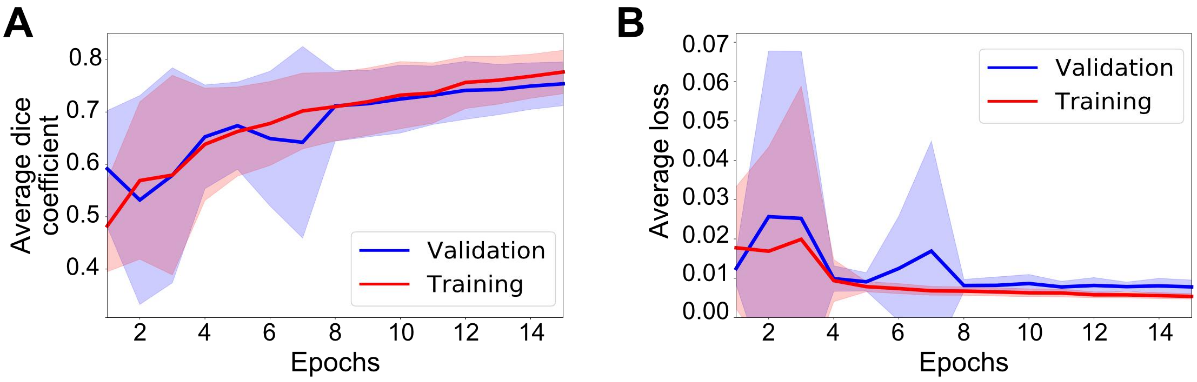

Figure S5: Average training performance of 12 different MitoSegNet models trained on 11 images for cross validation, Related to Figure 1. (A) Average validation (blue) and training (red) dice coefficient steadily increases over 15 epochs of training. **(B)** Average training loss decreases as expected, while validation loss enters a plateau after 8 epochs. Blue and red areas indicate the standard deviation added (upper border) or subtracted (lower border) from the average of the validation and training metric respectively.

Transparent Methods

General C. elegans methods and strains

C. elegans strains were cultured on Nematode Growth Medium (NGM) in petri plates (Brenner 1974). All experiments were carried out at 20°C and all strains were maintained at 20°C. *C. elegans* mitochondria in body wall muscle cells were labelled using an integrated transgene (*bcI78*) expressing mitochondrial matrix-targeted GFP under the control of the body wall muscle-specific promoter *myo-3* (P_{myo3}*::mitoGFP*) (Rolland, Motori et al. 2013). The following alleles were used LGII: *fzo-1(tm1133)* (National BioResource Project); *eat-3(ad426)* (Kanazawa, Zappaterra et al. 2008); LGIV: *drp-1(tm1108)* (National BioResource Project) and *catp-6(ok3473)* (Lambie, Tieu et al. 2013).

Generating the MitoSegNet model

The MitoSegNet model was trained for 20 epochs with 12 fluorescence microscopy images (1300 x 1030 px), depicting adult *C. elegans* mitochondria (tubular, elongated, fragmented and mixed) in body wall muscle cells labelled with mitoGFP (Fig. 1A). The images were recorded on a fluorescence microscope equipped with a 63x 1.4 NA oil lens (Axioskop 2; Carl Zeiss Inc.) and a charge-coupled device camera (1300; Micromax). Two expert annotators manually segmented a set of 6 microscopy images. The image annotations were generated by drawing separate regions of interest over all mitochondria within one image and then converting the selections into a binary mask. Raw 3D microscopy images were converted to 2D images through maximum intensity projection. Based on the expert annotation of four mitochondrial phenotypes (elongated, fragmented, mixed and tubular), 3 images of each phenotype were used for training. Prior to augmentation each image was divided into four overlapping 656 x 656 px images due to GPU memory constraints. The

input tile size was chosen to allow for the 2x2 max-pooling operations during training. Each image (and the corresponding ground truth) underwent random augmentation using the Keras library, producing 80 differently augmented images per input image, which involved random shearing (in a range of 30%), rotations (in a range of 180°), change of zoom (in a range of 30%), brightness change (in a range of 20%), horizontal and vertical flipping, x- and y-shifts (in a range of 20%) and mirroring. This increased the total number of images used for training from 48 to 3,840. We used a batch size of 1 and included the weight map as implemented in the U-Net publication and set w_{bal} to 0.042 (1 dived by the number of background pixels per object pixel to achieve a 1:1 ratio between object and background pixels). The MitoSegNet model was trained at a learning rate of $7*10^{-5}$ for 20 epochs and reached its minimum validation loss at epoch 18 (Fig. S4B).

MitoSegNet model prediction

To evaluate the segmentation performance of the MitoSegNet model on all 12 images, we performed a cross validation and generated 12 separately trained models (in the same manner as described above) (Fig. S5). Each model was trained with 11 images for 15 epochs and applied each trained MitoSegNet model to the test image that was excluded from training (Fig. 1B). Training and prediction were performed using our Python-based MitoS tool on a Devuan GNU/Linux server with an Nvidia TITAN X (12 GB GDDR5 RAM), using CUDA 10.0 and cuDNN 7.6.1.

The MitoS segmentation and MitoA analysis tool

The MitoS tool is a Python-based, standalone executable application that can be used for deep learning based segmentation of 2-dimensional microscopy images. The tool can be

executed in two modes: the "basic mode" is designed for researchers with no prior deep learning experience (Fig. 1), the "advanced mode" is intended for people with such experience and allows customizing training parameters (Fig. S1). For those users that do not have a CUDA-capable GPU, a CPU-only version of the MitoS segmentation tool is available.

The MitoS basic mode

uses the pretrained mitochondria-specific MitoSegNet model and any images depicting mitochondria images similar to those used for training can be segmented with the MitoS tool (Fig. 1). If the MitoSegNet model segmentation does not yield satisfactory results, self-generated training data can be used to finetune the existing segmentation model (Fig. S1A). Because the MitoSegNet model was generated using 656x656 px image tiles, any images intended for segmentation are fitted to this pre-set tile size. Images larger than the pre-set tile size are split into overlapping tiles with a mirrored border to avoid prediction in border regions. Smaller images are not split but instead a mirrored border is added to increase the size to the pre-set tile size. After prediction the mirrored parts of the tiles are removed and the tiles are stitched back together. A final threshold is applied to the fully stitched image to convert it to an 8-bit binary mask ($p_i < 128$ to 0 and $p_i >= 128$ to 255). Furthermore, any objects below 10 px in size are considered to be noise and are thus removed from the final segmentation image. The finetuning module automatically generates weight maps and augments the novel training data using the following augmentation operations: shearing (in a range of 30%), rotation (in range of 180°), zoom (in a range of 30%) and brightness change (in a range of 20%), horizontal and vertical flip, width and height shift (in a range of 20%). Once augmentation is completed, model training begins with a pre-set learning rate of 0.0001, a batch size of 1 and a class balance factor of 1/(foreground to background pixel ratio). We tested the MitoS finetuning module on images depicting adult *C. elegans* mitochondria visualised with a non-integrated P_{myo-3}*::mitoGFP* reporter (Rolland, Motori et al. 2013). The

intensity of the fluorescently labelled mitochondria appeared weaker and hence the MitoSegNet model failed to accurately segment these images (Fig. S2). One image of weak fluorescent intensity mitochondria was therefore manually segmented and used to finetune the pretrained model for 10 epochs, generating a finetuned MitoSegNet model that generated satisfactory segmentation results (Fig. S2). Alternatively, it is possible to create a custom segmentation model using the advanced mode of the MitoS tool (Fig. S1B). The advanced mode consists of four modules: Module 1 automatically generates a folder structure that is later used for model training. Module 2 enables data augmentation, which allows the user to specify the range of the above listed augmentation operations. Module 3 carries out the model training for which the parameters can be freely chosen. Module 4 predicts new segmentations using the fully trained model (Fig. S2).

The MitoA analyser tool is a separate Python-based application that can be run after successful segmentation for quantification and visualisation of potential morphological differences (Fig. S3). It measures 13 different features using both raw and segmented image for each object: area, minor and major axis length, eccentricity, perimeter, solidity, mean, max and min intensity, number of branches, branch length, total branch length and curvature index. The minor or major axis lengths are defined as the lengths of the line segment connecting the two co-vertices or vertices of an ellipse fitted around an object. The eccentricity of a conic section is a non-negative real number that characterizes its shape and the eccentricity for a circle, ellipse and parabola are 0, $0 > x > 1$ or 1 respectively. The solidity is defined as the object area divided by the convex object area. The curvature index indicates if the branches of the mitochondria are straight or curved and is defined as

$$c_i = \frac{branch\ length - euclidean\ distance}{euclidean\ distance}$$

The Euclidean distance is the shorted path between the start and end point of one branch. The single object features are summarised for each image as average, median, standard deviation, standard error, minimum and maximum and saved in an Excel table. One table is generated for a group of images, which can subsequently be compared. Both the two- and multi-comparison of samples allows to generate a table containing statistically relevant information and a visualisation using boxplots. Each statistical table contains the descriptors p-value of the D'Agostino's K-squared test to determine if the data is normally distributed (D'Agostino 1971, D'Agostino and Pearson 1973). Based on this result and the Levene's test p-value to test for equality of variances an appropriate hypothesis test is chosen and the name of the test, including its p-value are displayed in the table (Levene 1960). For comparison of two normally distributed datasets with equal variance a Student's t-test is used . If either of the two criteria are not met, the Mann–Whitney U test is used. For comparison of multiple samples with equal variance and normal distributions, a one-way ANOVA is used or alternatively, the Kruskal-Wallis-Test is applied (Kruskal and Wallis 1952). For a two-sample comparison the effect size is also calculated, and the boxplot visualisation indicates statistically significant differences. Furthermore, the two-sample comparison also enables the user to perform a correlation analysis. The correlation analysis visualises correlation by letting the user select up to four different descriptors to correlate against each other in both samples and then have them displayed as scatterplots. The distributions are subjected to the D'Agostino's K-squared test to determine the usage of the Pearson or Spearman rank-order correlation. Correlation coefficients and p-values are displayed in the MitoA terminal or can be saved to an Excel file. The MitoSegNet Analyser was used for statistical testing and visualisation of morphological differences for Figure 4 and 5.

Both tools have been tested internally on Windows and Linux platforms and will be actively maintained on GitHub.

The MitoSegNet architecture

The MitoSegNet architecture is based on the previously published U-Net (Ronneberger, Fischer et al. 2015) and was implemented in Keras (Python 3.7.3). It consists of a contracting path, which follows the standard architecture of convolutional neural networks, consisting of repeated application of 3x3 convolutions (padded convolutions), each followed by a batch normalization layer, a rectified linear unit (ReLU) and a 2x2 max pooling operation with a stride of 2. Unlike the original U-Net, the MitoSegNet does not utilize any drop-out layers at the end of the contracting path as we have found the batch normalization layer to reduce the training time. After 1024 feature channels have been generated, the expanding pathway uses 2x2 up-convolutions to halve the number of channels, followed by a concatenation with the corresponding feature map from the contracting path and subsequent 3x3 convolutions, followed by a ReLU. The final convolutional layer (1x1) is followed by a sigmoid function. In total, the MitoSegNet consists of 24 convolutional layers. The optimization algorithm chosen for the training process is Adam (adaptive moment estimation), which is an extension to stochastic gradient descent (Diederik P. Kingma 2014). During training, 20% of the data is excluded from the process and instead used for model validation after each training epoch. The energy function is computed by a pixel-wise sigmoid function over the final feature map combined with a binary cross entropy loss function

$$p_k(x) = \frac{1}{1 + e^{-a_k(x)}}$$

where $a_k(x)$ denotes the activation in feature channel k at the pixel position x and $p_k(x)$ is the approximated maximum-function for that feature channel. The binary cross entropy is then used to calculate the loss at each pixel

$$E = -\sum_{i=1}^{2} w \cdot y_i^{'} \cdot \log(y_i)$$

where y_i is the predicted probability of the class i, $y_i^{'}$ is the true probability for that class and w is the weight map. The separation weight map w_{sep} is implemented as described in (Ronneberger, Fischer et al. 2015) and prevents touching objects to be segmented as one object by increasing the weights in border regions. The MitoSegNet also includes a class balancing weight map, which decreases the weight of background pixels by a factor of w_{bal} so the final weight map is defined as

$$w = w_{bal} + w_{sep}$$

Just as in the original U-Net, initial weights are drawn from a Gaussian distribution with a standard deviation of $\sqrt{2/N}$, where N is the number of incoming nodes of one neuron.

Other segmentation methods

All segmentation methods include the removal of uninformative image slices from the stack and the remaining stack is reduced to a single image via maximum intensity projection. Prior to all Fiji (ImageJ) segmentation methods a background subtraction (rolling ball algorithm with a radius of 15 pixels) is applied (Schneider, Rasband et al. 2012). After applying the three different feature enhancement approaches with Fiji, they are all followed by a final filter step in which all particles smaller than 10 px in size are removed from the final mask.

The method denoted as **Laplacian** is based on the de Boer lab workflow on quantitative analysis of mitochondrial morphology in *C. elegans* (de Boer, Smith et al. 2015). It is based on the application of a local contrast enhancement (CLAHE, in Fiji with a block size = 15, histogram = 256, maximum = 3) and subsequent object enhancement using a multi-scale Laplacian operator (FeatureJ Laplacian in Fiji with a compute smoothing setting of 1.0) (De Vos, Van Neste et al. 2010). The images are then binarized according to a Yen autothresholding procedure (Yen, Chang et al. 1995).

The **Hessian** method calculates eigenvalues of a Hessian matrix using the Tubeness plugin in Fiji with a sigma value of 1.0 (Sato, Nakajima et al. 1998). To generate a binary image the IsoData autothresholding is used (Huang and Wang 1995).

The **Gaussian** method calculates the difference of Gaussians by generating two gaussian blurred versions of the original image and subtracting the blurred image with the higher sigma (σ= 4) from the image with lower sigma (σ= 2). The binary mask is generated using default autothresholding.

To also include a machine learning segmentation approach we used the open-source software **Ilastik**. This software learns from labels provided by the user, using a random forest classifier in the learning step, in which each pixel's neighbourhood is characterized by a set of nonlinear features (Kreshuk and Zhang 2019). To train the classifier, 12 images were used for training. Each appendant label was created with ilastik by partially marking objects of interest and background.

Segmentation performance on test set

We evaluated the segmentation performance of the six approaches with different measures, each focusing on particular aspects of the segmentation result with specific limitations. The Dice coefficient (dc, also known as F1 score) (Dice 1945, Sørensen 1948) is a statistic value used for comparing the similarity of two binary datasets.

$$dc = \frac{2 \cdot |ground\ truth\ \cap\ prediction|}{|ground\ truth| + |prediction|}$$

On each image, the dc is evaluated on all pixels. The distributions of dice coefficients from all images were tested for normality using the D'Agostino's K-squared test and statistical difference was determined using the Kruskal-Wallis test followed by Dunn's multiple comparisons test. Upon comparing the dc's with the visual segmentation results, we realised that the dc values do not fully reflect the morphological segmentation accuracy (see Fig. 4B).

To obtain information about morphological segmentation accuracy, we compared single object shapes. The single object shape comparison uses the following shape descriptors for comparison: area, eccentricity, aspect ratio (dividing the major axis length by the minor axis length), perimeter and solidity. The difference between predicted and ground truth shape descriptor is calculated as fold deviation for each object and defined as

$$sd_{dev} = \frac{|sd_p - sd_{gt}|}{sd_p}$$

where sd_p is the predicted and sd_{gt} the ground truth shape descriptor. Object correspondence was assumed if at least one identical pixel coordinate was found in both the ground truth and the predicted object. Only objects that were predicted to correspond with a single object in the ground truth or vice versa were included in this analysis. For single correspondence, a fold deviation (the predicted value multiplied or divided by that fold deviation would yield the ground truth value) for all five morphological descriptors from the ground truth is then

calculated for each object (Fig. 4C). The single object fold deviation values were tested for normality using the D’Agostino’s K-squared test and statistical differences between methods were determined using the Kruskal-Wallis test followed by Dunn’s multiple comparisons test. Because the single object shape comparison neglects false positive predictions, we compared the predicted shape distribution of all objects per image against the ground truth. The shape descriptors are the same as used for the single object shape comparison. For segmented objects, distributions of these five descriptors were obtained and compared to the distributions of the ground truth images by calculating the energy distance (Fig. 4E). The energy distance between two distributions d1 and d2 is defined as

$$D(d1, d2) = (2E\,|X - Y| - E|X - X'| - E|Y - Y'|)^{1/2}$$

where X and X' (resp. Y and Y') are independent random variables with a probability distribution of *d1* (resp. *d2*) (Szekely 2002). Energy distance values were normalised prior to being tested for normality using the D’Agostino’s K-squared test, followed by applying a Kruskal-Wallis test and a subsequent Dunn’s multiple comparisons test.

All methods to test the performance of each segmentation approach were implemented in Python 3.7.3 using the following libraries: NumPy, OpenCV, scikit-image, scikit-learn, scikit-posthocs, Matplotlib, Seaborn, Pandas and SciPy.

Comparison of mitochondrial morphology between *catp-6* mutant and wild-type

Images segmented with the pretrained segmentation model were recorded with a fluorescent microscope, using a 63x 1.4 NA oil lens (Axioskop 2; Carl Zeiss Inc.) and a charge-coupled device camera (1300; Micromax). The *C. elegans* strain carrying the *bcIs78* transgene and the

catp-6(ok3473) mutation was compared to the wild-type strain carrying the bcIs78 transgene. Quantitative analysis was performed using the MitoA tool.

Application of the MitoSegNet model segmentation to mitochondria in HeLa cells

Images segmented with the pretrained segmentation model were recorded with an inverted Zeiss LSM 880 system equipped with a DPSS 561-nm laser, using a Plan-Apochromat 63x / 1.4 oil DIC objective and a GaAsP detector. Images were collected with the ZEN 2 software at 1024 x 1024 pixels resolution. To visualize mitochondria, HeLa cells (ATCC) were transfected with mitoRFP using Lipofectamine 2000. After 24 hours of transfection, the HeLa cells were either left untreated or treated with 2.5 μM oligomycin (Calbiochem) and 1 μM antimycin A (Sigma) in fresh growth medium for 3 hours. Segmented and raw images were subjected to quantitative analysis using the MitoA tool.

References

Brenner, S. (1974). "The genetics of Caenorhabditis elegans." Genetics 77(1): 71-94.
D'Agostino, R. B. (1971). "An omnibus test of normality for moderate and large sample size." Biometrika 58: 341-348.

D'Agostino, R. B. and E. S. Pearson (1973). "Tests for departure from normality." Biometrika 60: 613-622.

de Boer, R., R. L. Smith, W. H. De Vos, E. M. Manders, S. Brul and H. van der Spek (2015). "Caenorhabditis elegans as a Model System for Studying Drug Induced Mitochondrial Toxicity." PLoS One 10(5): e0126220.

De Vos, W. H., L. Van Neste, B. Dieriks, G. H. Joss and P. Van Oostveldt (2010). "High content image cytometry in the context of subnuclear organization." Cytometry Part A 77A(1): 64-75.

Dice, L. R. (1945). "Measures of the Amount of Ecologic Association Between Species." Ecology 26(3): 297-302.

Diederik P. Kingma, J. B. (2014). "Adam: A Method for Stochastic Optimization." CoRR abs/1412.6980.

Huang, L. and M. Wang (1995). "Image thresholding by minimizing the measure of fuzziness." Pattern Recognition 28(1): 41-51.

Kanazawa, T., M. D. Zappaterra, A. Hasegawa, A. P. Wright, E. D. Newman-Smith, K. F. Buttle, K. McDonald, C. A. Mannella and A. M. van der Bliek (2008). "The C. elegans Opa1 Homologue EAT-3 Is Essential for Resistance to Free Radicals." PLOS Genetics 4(2): e1000022.

Kreshuk, A. and C. Zhang (2019). "Machine Learning: Advanced Image Segmentation Using ilastik." Methods Mol Biol 2040: 449-463.

Kruskal, W. H. and W. W. Wallis (1952). "Use of Ranks in One-Criterion Variance Analysis." Journal of the American Statistical Association 47(260): 583-621.

Lambie, E. J., P. J. Tieu, N. Lebedeva, D. L. Church and B. Conradt (2013). "CATP-6, a C. elegans ortholog of ATP13A2 PARK9, positively regulates GEM-1, an SLC16A transporter." PLoS One 8(10): e77202.

Levene, H. (1960). "Robust tests for equality of variances. In Ingram Olkin; Harold Hotelling; et al. (eds.). Contributions to Probability and Statistics: Essays in Honor of Harold Hotelling." Stanford University Press: 278-292.

Rolland, S. G., E. Motori, N. Memar, J. Hench, S. Frank, K. F. Winklhofer and B. Conradt (2013). "Impaired complex IV activity in response to loss of LRPPRC function can be compensated by mitochondrial hyperfusion." Proc Natl Acad Sci U S A 110(32): E2967-2976.

Ronneberger, O., P. Fischer and T. Brox (2015). U-Net: Convolutional Networks for Biomedical Image Segmentation, Cham, Springer International Publishing.

Sato, Y., S. Nakajima, N. Shiraga, H. Atsumi, S. Yoshida, T. Koller, G. Gerig and R. Kikinis (1998). "Three-dimensional multi-scale line filter for segmentation and visualization of curvilinear structures in medical images." Med Image Anal 2(2): 143-168.

Schneider, C. A., W. S. Rasband and K. W. Eliceiri (2012). "NIH Image to ImageJ: 25 years of image analysis." Nat Methods 9(7): 671-675.

Sørensen, T. (1948). "A method of establishing groups of equal amplitude in plant sociology based on similarity of species and its application to analyses of the vegetation on Danish commons." Kongelige Danske Videnskabernes Selskab 5(4): 1-34.

Szekely (2002). "E-statistics: The energy of statistical samples." Technical Report 02-16.

Yen, J., F. Chang and S. Chang (1995). "A New Criterion for Automatic Multilevel Thresholding." IEEE Trans. on Image Processing 4(3): 370-378.
